# Endosymbiont-based immunity in *Drosophila melanogaster* against parasitic nematode infection

**DOI:** 10.1371/journal.pone.0192183

**Published:** 2018-02-21

**Authors:** Shruti Yadav, Joanna Frazer, Ashima Banga, Katherine Pruitt, Sneh Harsh, John Jaenike, Ioannis Eleftherianos

**Affiliations:** 1 Infection and Innate Immunity Lab, Department of Biological Sciences, George Washington University, Washington, District of Columbia, United States of America; 2 Thomas Jefferson High School for Science and Technology, Alexandria, Virginia, United States of America; 3 Department of Biology, University of Rochester, Rochester, New York, United States of America; University of Innsbruck, AUSTRIA

## Abstract

Associations between endosymbiotic bacteria and their hosts represent a complex ecosystem within organisms ranging from humans to protozoa. *Drosophila* species are known to naturally harbor *Wolbachia* and *Spiroplasma* endosymbionts, which play a protective role against certain microbial infections. Here, we investigated whether the presence or absence of endosymbionts affects the immune response of *Drosophila melanogaster* larvae to infection by *Steinernema carpocapsae* nematodes carrying or lacking their mutualistic Gram-negative bacteria *Xenorhabdus nematophila* (symbiotic or axenic nematodes, respectively). We find that the presence of *Wolbachia* alone or together with *Spiroplasma* promotes the survival of larvae in response to infection with *S*. *carpocapsae* symbiotic nematodes, but not against axenic nematodes. We also find that *Wolbachia* numbers are reduced in *Spiroplasma*-free larvae infected with axenic compared to symbiotic nematodes, and they are also reduced in *Spiroplasma*-containing compared to *Spiroplasma*-free larvae infected with axenic nematodes. We further show that *S*. *carpocapsae* axenic nematode infection induces the Toll pathway in the absence of *Wolbachia*, and that symbiotic nematode infection leads to increased phenoloxidase activity in *D*. *melanogaster* larvae devoid of endosymbionts. Finally, infection with either type of nematode alters the metabolic status and the fat body lipid droplet size in *D*. *melanogaster* larvae containing only *Wolbachia* or both endosymbionts. Our results suggest an interaction between *Wolbachia* endosymbionts with the immune response of *D*. *melanogaster* against infection with the entomopathogenic nematodes *S*. *carpocapsae*. Results from this study indicate a complex interplay between insect hosts, endosymbiotic microbes and pathogenic organisms.

## Introduction

The soil dwelling nematode parasite *Steinernema carpocapsae* together with the Gram-negative bacteria *Xenorhabdus nematophila* form a mutualistic complex that is pathogenic to insects [1]. *X*. *nematophila* bacteria are localized in the gut of *S*. *carpocapsae* nematodes, which complete their life cycle in insect hosts [2]. The nematodes cause infections at the infective juvenile (IJ) stage, which is the developmentally arrested third larval stage analogous to the dauer stage of the non-pathogenic nematode, *Caenorhabditis elegans* [3]. Upon entry into the insect host, the nematodes release their bacteria into the hemolymph (insect blood), where the latter divide and produce a wide range of toxins and virulence factors that kill the host [4,5]. Although little is known about the contribution of nematode virulence factors to this process, we and others have shown that entomopathogenic (or insect pathogenic) nematodes lacking their mutualistic bacteria are still pathogenic to insects [6–10]. Recent studies have demonstrated that the nematodes produce certain molecules that suppress or promote evasion of certain insect immune responses allowing them to survive and reproduce in the insect host [11–13].

Insects have developed a diverse range of immune defenses to combat infection by nematode parasites [14]. Most studies have mainly focused on the immune response of insect larvae against entomopathogenic nematodes and the immune response of mosquitoes and black flies against filarial nematodes [9,10,14–17]. Insects activate both humoral and cellular immune responses to nematode infections as well as phenoloxidase (PO) and coagulation cascades that lead to melanotic encapsulation of the parasites [10,18–20]. Certain entomopathogenic nematodes have developed strategies to evade or suppress the insect immune system by preventing or disrupting the activation of immune responses to promote their survival in the host [14,21–23]. The fruit fly *Drosophila melanogaster* is an outstanding model for innate immunity studies. Its major benefit is the availability of a wide range of genetic tools that permit dissection of the molecular basis of the innate immune response to a range of pathogens [24–26]. Recent transcriptomic studies have demonstrated the power of using *D*. *melanogaster* for identifying the molecular components of the insect immune system that are directed against entomopathogenic nematode infections [9,15]. It was recently shown that *Steinernema* nematodes are able to upregulate the expression of certain antimicrobial peptide (AMP) genes and induce the melanization pathway, the activation of which is suppressed by *Xenorhabdus* bacteria [10].

*Wolbachia* and *Spiroplasma* are the most common and widespread maternally-transmitted facultative endosymbiotic bacteria in insects, and they are naturally harbored by certain *D*. *melanogaster* strains [27–30]. Recent studies have led to the proposition of endosymbiont-based strategies for the control of vector borne diseases [31–35]. *D*. *melanogaster* is an excellent system to investigate the effect of endosymbionts on host immune function. Previous studies have shown that the presence of certain *Wolbachia* strains in *D*. *melanogaster* confers resistance to infection by various RNA viruses, fungi and parasitoid wasps [36–41], but not by entomopathogenic bacteria [42–45]. The presence of *Spiroplasma* endosymbionts in *D*. *melanogaster* flies does not activate the fly immune system, but induction of Toll or immune deficiency (Imd) immune signaling increases *Spiroplasma* numbers in the fly hemolymph [46]. Furthermore, mushroom-feeding flies *D*. *neotestacea* carrying *Spiroplasma* have increased tolerance against their natural nematode parasite *Howardula aoronymphium* [47], which is probably due to an unknown mechanism that reduces the growth and reproduction of the nematodes in the *Spiroplasma*-carrying flies. Alternatively, flies carrying *Spiroplasma* are more sensitive to some Gram-negative bacterial pathogens [42, 46].

The goal of this research is to investigate whether the presence of heritable endosymbiont *Wolbachia* alone or together with *Spiroplasma* can modulate the *D*. *melanogaster* immune and metabolic response against *S*. *carpocapsae* nematodes that either carry (symbiotic) or lack (axenic) their associated *X*. *nematophila* bacteria. For this, we use *D*. *melanogaster* strains with or without their heritable endosymbionts for infections with *S*. *carpocapsae* symbiotic or axenic nematodes. We explore certain aspects of the immune response and estimate levels of triglyceride, glucose, trehalose, and glycogen in all *D*. *melanogaster* strains in the presence or absence of nematode infection. We also investigate the involvement of lipid droplets in the *D*. *melanogaster* anti-nematode immune response in the context of host endosymbionts. We find that the presence of *Wolbachia* in *D*. *melanogaster* larvae enhances the survival ability against *S*. *carpocapsae* symbiotic nematodes, *Wolbachia* numbers are reduced in larvae responding to symbiotic nematodes while *Xenorhabdus* numbers are unaffected, the absence of *Wolbachia* induces Toll pathway activation in response to axenic nematodes, and that endosymbionts can affect the metabolic state, and in particular the lipid droplet size, of *D*. *melanogaster* during parasitic nematode infection. Current findings reveal that *Wolbachia* and *Spiroplasma* interact closely with the *D*. *melanogaster* immune system and are able to modulate certain aspects of the larval response to infection against a potent nematode parasite.

## Results

### Presence of *Wolbachia* in *Drosophila* enhances the survival response to symbiotic nematode infection

**Fig 1:**
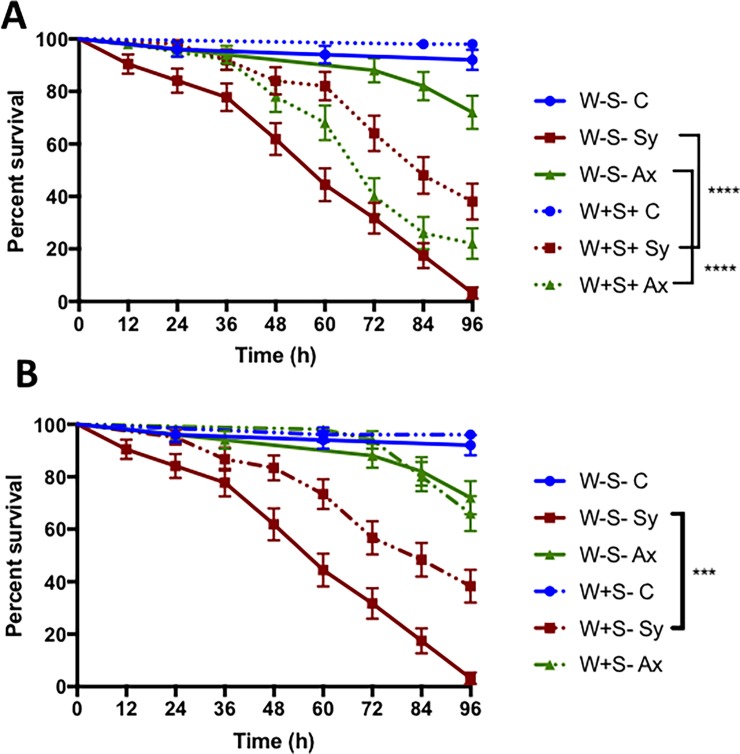
Survival of *Drosophila melanogaster* larvae carrying or lacking endosymbionts in response to nematode infection. Survival of *D*. *melanogaster* third-instar larvae upon infection with *Steinernema carpocapsae* symbiotic (Sy) or axenic (Ax) nematodes. Sterile distilled water served as control (C) treatment. (A) Survival response of *D*. *melanogaster* strains lacking both *Wolbachia* and *Spiroplasma* (W-S-) and strains carrying both endosymbionts (W+S+), (B) Survival response of *D*. *melanogaster* strains lacking both endosymbionts (W-S-) and strains carrying *Wolbachia* only (W+S-). Survival was tracked every 12 h for 96 h and is represented as percent survival on the graph. Data were analyzed using the Log-Rank test (GraphPad Prism7 software). The experiment was repeated three times and bars represent standard errors (****P<0.001, ****P<0.0001).

We first estimated the survival ability of *D*. *melanogaster* W+S-, W+S+ and W-S- larvae in response to *S*. *carpocapsae* symbiotic or axenic nematodes. We found significant differences in the survival between each *D*. *melanogaster* strain infected by either symbiotic or axenic nematodes and the uninfected controls (Fig 1). We also found that W+S+ larvae infected with symbiotic nematodes survived significantly better than W-S- larvae (Log-rank test, *P*<0.0001; Fig 1A), and this result was reversed upon infection with axenic nematodes (log-rank test, *P*<0.0001; Fig 1A). We further observed that the W+S- larvae survived the infection with symbiotic nematodes longer than the W-S- individuals (log-rank test, *P*<0.0001; Fig 1B), but there were no statistically significant differences in survival between W-S- and W+S- larvae upon infection with axenic nematodes (log-rank test, *P* = 0.6154, Fig 1B). These results suggest that the presence of *Spiroplasma* does not affect the survival of *D*. *melanogaster* against symbiotic *S*. *carpocapsae* when *Wolbachia* is also present; however, the presence of *Spiroplasma* together with *Wolbachia* is detrimental to the larvae upon axenic nematode infection.

### *Wolbachia* numbers are reduced in *Drosophila* responding to symbiotic nematodes while *Xenorhabdus* numbers are unaffected

**Fig 2:**
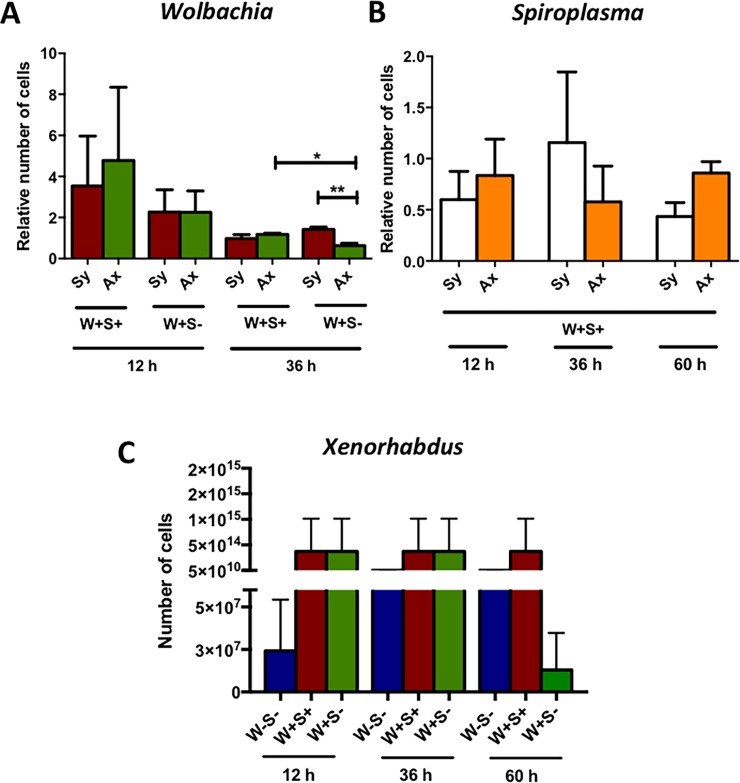
Numbers for endosymbiotic and pathogenic bacteria in *Drosophila melanogaster* larvae responding to nematode infection. *D*. *melanogaster* third instar larvae carrying no endosymbionts (W-S-), both endosymbionts (W+S+) or only *Wolbachia* (W+S-) were infected with *Steinernema carpocapsae* symbiotic (Sy) or axenic (Ax) nematodes. Relative number of cells for (A) *Wolbachia* at 12 and 36 h, and (B) *Spiroplasma* at 12, 36 and 60 h were determined using quantitative PCR. (C) Numbers of colony forming units of *Xenorhabdus nematophila* were estimated at 12, 36 and 60 h post infection using quantitative PCR. Data were analyzed using an unpaired two-tailed t-test. Means from three independent experiments are shown and standard deviations are represented by error bars (*P<0.05, **P<0.01).

To estimate whether infection by *S*. *carpocapsae* nematodes affects the numbers of endosymbiotic bacteria in *D*. *melanogaster*, we infected W+S-, W+S+ and W-S- larvae with *S*. *carpocapsae* symbiotic or axenic nematodes and estimated the endosymbiont numbers at different time points post infection. Interestingly, *Wolbachia* numbers were significantly reduced in W+S- larvae infected with axenic nematodes compared to symbiotic nematodes at 36 h post-infection (*P* = 0.0076; Fig 2A), and they were also significantly lower than those in W+S+ larvae infected with axenic nematodes at 36 h post infection (*P* = 0.0143; Fig 2A). There were no significant changes in the numbers of *Spiroplasma* in W+S+ larvae infected by either symbiotic or axenic nematodes at any time-point (*P*>0.05; Fig 2B). These results imply that the presence of *X*. *nematophila* bacteria in *S*. *carpocapsae* and the presence of *Spiroplasma* in *D*. *melanogaster* larvae can affect the number of *Wolbachia* endosymbionts at late times after infection with the nematode parasites.

To estimate whether *X*. *nematophila* replication is affected in the presence or absence of the endosymbionts in *D*. *melanogaster*, we infected W-S-, W+S+ and W+S- larvae with *S*. *carpocapsae* symbiotic nematodes and estimated the *X*. *nematophila* numbers at three time-points post infection. We found no significant differences in the number of *X*. *nematophila* Colony Forming Units (CFUs) in W+S+ larvae between the different time-points post infection (*P*>0.05; Fig 2C). In W-S- larvae, the increase in *X*. *nematophila* CFUs was not statistically different (*P*>0.05; Fig 2C). In addition, larvae carrying only *Wolbachia* (W+S-) contained fewer *X*. *nematophila* CFUs at 60 h post symbiotic nematode infection compared to 12 and 36 h, but again no statistically significant difference was observed (*P*>0.05; Fig 2C). These results suggest that the presence of *Wolbachia* and *Spiroplasma* endosymbionts in *D*. *melanogaster* does not have a significant impact on *X*. *nematophila* load during *S*. *carpocapsae* nematode infection.

### Absence of *Wolbachia* in *Drosophila* can induce Toll pathway activation in response to axenic nematode infection

**Fig 3:**
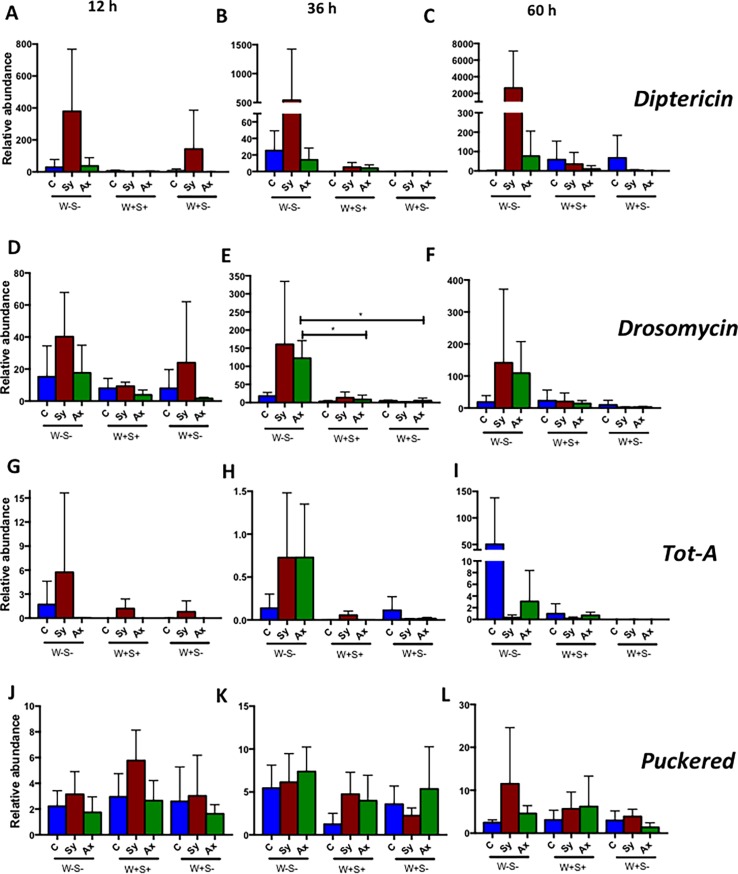
Transcript levels of immune genes in *Drosophila melanogaster* larvae carrying or lacking endosymbionts upon nematode infection. Gene transcript levels for (A, B and C) *Diptericin*, (D, E and F) *Drosomycin*, (G, H and I) *Turandot-A* (*Tot-A*), and (J, K and L) *Puckered* in *D*. *melanogaster* larvae containing no endosymbionts (W-S-), both *Wolbachia* and *Spiroplasma* (W+S+), or *Wolbachia* only (W+S-) at 12, 36 and 60 h after infection with *Steinernema carpocapsae* symbiotic (Sy) or axenic (Ax) nematodes. Sterile distilled water served as control (C) treatment. The experiment was repeated three times and error bars show standard deviations. Data were analyzed using one way analysis of variance with a Tukey post *hoc* test (*P<0.05).

The transcriptional activation of immune signaling pathway read-out genes forms the hallmark of the *D*. *melanogaster* humoral immune response. Here, we followed this approach to examine immune signaling pathway activation upon infection with *S*. *carpocapsae* symbiotic or axenic nematodes in the presence or absence of endosymbionts (Fig 3). For this, we used real-time quantitative reverse transcription polymerase chain reaction (qRT-PCR) and gene-specific primers to determine the transcript levels of *Diptericin* as a readout for the Imd pathway, *Drosomycin* for the Toll pathway, *Turandot A* (*TotA*) for the Jak/Stat pathway and *Puckered* for the Jnk pathway [48–51]. We found that induction of *Diptericin* was higher in W-S- larvae infected with symbiotic nematodes at all three time points post infection, but this induction was not statistically significant compared to axenic nematode infections (Fig 3A, 3B and 3C). We also found no changes in *Drosomycin* transcript levels between the different strains at 12 and 60 h post nematode infection (*P*>0.05; Fig 3D and 3F), but *Drosomycin* transcript levels were significantly higher in W-S- larvae than in W+S+ and W+S- individuals infected with axenic nematodes at 36 h (*P* = 0.0165 and *P* = 0.0141, respectively) and compared to control uninfected larvae (*P* = 0.0212; Fig 3E). There were no significant differences in *TotA* and *Puckered* transcript levels among the three *D*. *melanogaster* strains infected by either symbiotic or axenic nematodes compared to uninfected controls (*P*>0.05; Fig 3G–3I and 3J–3L). These results suggest that the absence of *Wolbachia* endosymbionts in *D*. *melanogaster* larvae can activate Toll signaling in the context of axenic nematode infection.

### Endosymbionts do not affect the PO response to nematode infection in *Drosophila*

**Fig 4:**
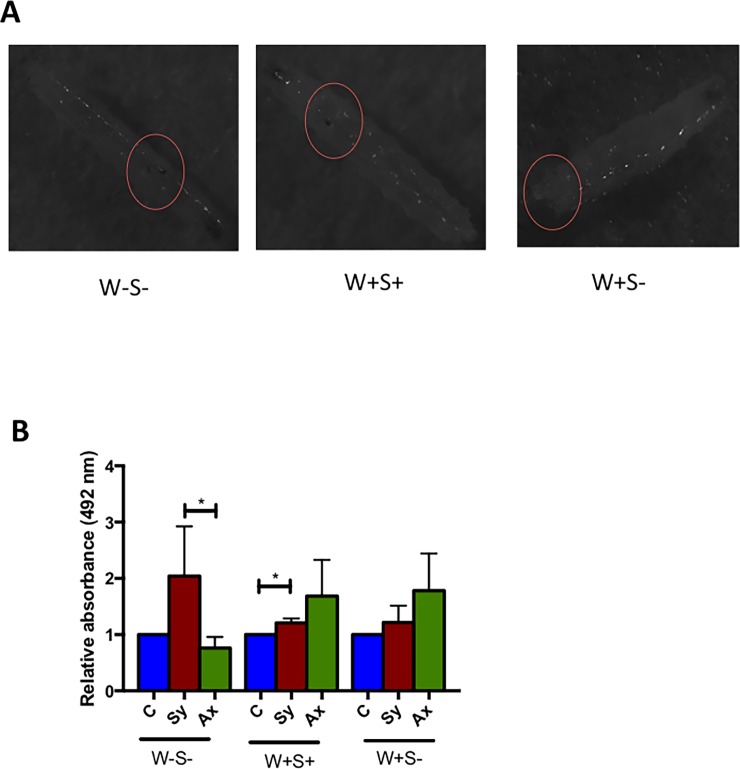
Phenoloxidase activity and melanization response in uninfected and nematode-infected *Drosophila melanogaster* larvae carrying or lacking endosymbionts. (A) Melanization response in *D*. *melanogaster* larvae containing no endosymbionts (W-S-), both *Wolbachia* or *Spiroplasma* (W+S+), or *Wolbachia* only (W+S-) following heat treatment. (B) Relative phenoloxidase (PO) activity was measured in the larval hemolymph of the three *D*. *melanogaster* strains at 24 h post-infection with *Steinernema carpocapsae* symbiotic (Sy) or axenic (Ax) nematodes. Sterile distilled water served as control (C) treatment. The experiment was repeated three times and error bars show standard deviations. Data analysis was performed using one way analysis of variance with a Tukey post *hoc* test (*P<0.05).

We first examined the melanization response of each *D*. *melanogaster* strain carrying or lacking endosymbionts after heat treatment [52], and observed that larvae developed dark spots indicating PO activation in crystal cells (Fig 4A). Upon nematode infection, PO activity in W-S- larvae was significantly higher in response to symbiotic nematodes compared to axenic nematodes and uninfected controls (*P* = 0.0392; Fig 4B). In W+S+ larvae, PO activity was also significantly higher upon symbiotic nematode infection compared to uninfected larvae (*P* = 0.0111; Fig 4B). However, in W+S- larvae, PO activity was higher upon infection with axenic nematodes compared to symbiotic nematode infections, but this difference was not statistically significant (*P* = 0.9972; Fig 4B). These results suggest that *S*. *carpocapsae* symbiotic nematode infection in *D*. *melanogaster* larvae can induce PO activity, which is not significantly affected by the presence or absence of endosymbiotic bacteria.

### Endosymbionts can alter the metabolic state of *Drosophila* upon nematode infection

**Fig 5:**
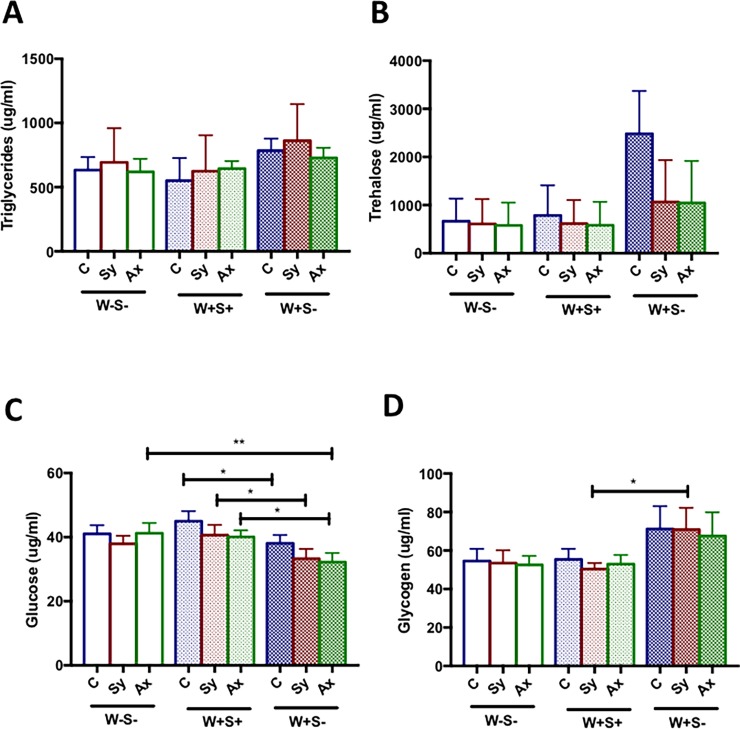
Metabolic activity in *Drosophila melanogaster* larvae carrying or lacking endosymbionts following nematode infection. *D*. *melanogaster* third instar larvae lacking both endosymbionts (W-S-), carrying both *Wolbachia* and *Spiroplasma* (W+S+), or containing *Wolbachia* only (W+S-) were infected with *Steinernema carpocapsae* symbiotic (Sy) or axenic (Ax) nematodes. Application of sterile distilled water served as control (C) treatment. The relative amount of (A) Triglycerides, (B) Trehalose, (C) Glucose and (D) Glycogen was estimated 24 h post-infection. The experiment was repeated three times and error bars show standard deviations. Data were analyzed using one way analysis of variance with a Tukey post *hoc* test (*P<0.05, **P<0.01).

To estimate whether the presence or absence of endosymbionts affects the metabolic functions of *D*. *melanogaster* in response to nematode infection, we infected W-S-, W+S+ and W+S- larvae with *S*. *carpocapsae* symbiotic or axenic nematodes and measured various metabolic processes 24 h post infection. We found that upon axenic or symbiotic nematode infection, changes in triglyceride concentrations were not statistically significant (*P*>0.05; Fig 5A). Also, trehalose levels were not statistically different among the various treatments (*P*>0.05; Fig 5B). Interestingly, the amount of glucose was significantly higher in W+S+ larvae compared to W+S- larvae upon infection with symbiotic or axenic nematodes (*P* = 0.0251 and *P* = 0.0469, respectively) as well as in uninfected controls (*P* = 0.0422; Fig 5C). We further found a significant increase in glucose levels in W-S- larvae compared to W+S- larvae when responding to axenic nematodes (*P* = 0.0032; Fig 5C). In the context of symbiotic nematode infection, the amount of glycogen in W+S- larvae was significantly higher than in W+S+ larvae (*P* = 0.0324; Fig 5D). These findings indicate that *Wolbachia* and *Spiroplasma* can affect glucose and glycogen levels in *D*. *melanogaster* larvae upon *S*. *carpocapsae* nematode infection, but have no effect on triglyceride or trehalose levels.

### Presence of both endosymbionts can alter lipid droplet size in *Drosophila* larvae responding to parasitic nematodes

**Fig 6:**
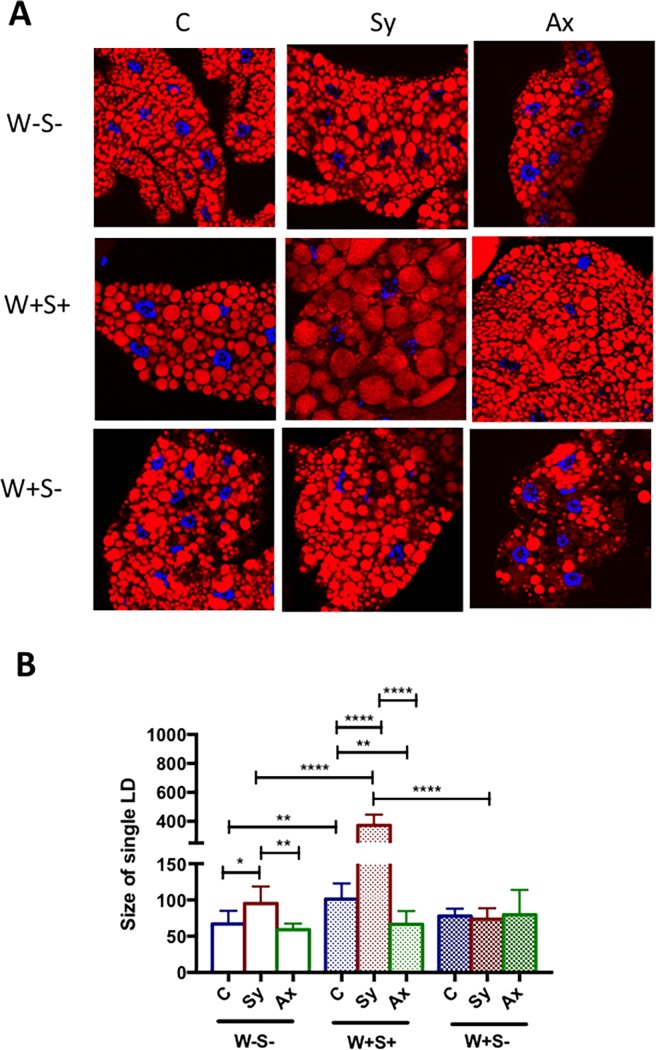
Lipid droplet size in *Drosophila melanogaster* larvae carrying or lacking endosymbionts upon nematode infection. (A) Representative images of lipid droplets (LD) labeled with Nile Red (red) and DAPI (blue) in fat body tissues of *D*. *melanogaster* third instar larvae lacking both endosymbionts (W-S-), containing both *Wolbachia* and *Spiroplasma* (W+S+), or carrying *Wolbachia* only (W+S-) followed infection with *Steinernema carpocapsae* symbiotic (Sy) or axenic (Ax) nematodes. Sterile distilled water served as control (C) treatment. Magnification: 40X. (B) Quantification of LD area in the fat body tissues obtained from 10 *D*. *melanogaster* larvae per treatment using ImageJ. Values show the means from three independent experiments and error bars show standard deviations. Data were analyzed using one way analysis of variance with a Tukey post *hoc* test (*P<0.05, **P<0.01, ****P<0.0001).

Recent studies have demonstrated an interaction between the host and pathogen metabolism. The supply of metabolites from the commensal bacteria to its host can be consumed by the pathogen, which leads to an increase in the lipid droplet size in the insect fat body [53]. Here, we evaluated whether the presence or absence of endosymbionts influences the size of lipid droplets in *D*. *melanogaster* during infection with entomopathogenic nematodes. We found that in uninfected individuals, the size of lipid droplets was significantly larger in W+S+ larvae compared to W-S- individuals (*P* = 0.0081; Fig 6A and 6B). We also found that W+S+ larvae contained lipid droplets of larger size upon infection with symbiotic nematodes compared to W-S- and W+S- larvae (*P*<0.0001) and to uninfected controls (*P*<0.0001; Fig 6A and 6B). In contrast, W+S+ larvae contained reduced size lipid droplets upon axenic nematode infection compared to controls (*P* = 0.0066; Fig 6A and 6B). The lipid droplet size in W+S- larvae was unaffected by nematode infection. These results suggest that the presence or absence of both endosymbionts might alter the lipid droplet size in response to *S*. *carpocapsae* axenic or symbiotic infections. On the contrary, the presence of *Wolbachia* alone has no effect on the size of lipid droplets upon nematode infection.

## Discussion

Previous studies in *D*. *melanogaster* adult flies have shown a protective role for *Wolbachia*, but not *Spiroplasma*, in response to certain viral infections [36,37], but not against bacterial infections [42,43,46,54]. Here, we explore the modulation of the *D*. *melanogaster* immune and metabolic responses, in the presence of *Wolbachia* alone or together with *Spiroplasma*, against *S*. *carpocapsae* nematodes. We find that the presence of *Wolbachia* alone or together with *Spiroplasma* in *D*. *melanogaster* larvae increases their survival upon infection with symbiotic *S*. *carpocapsae*; whereas the presence of both endosymbionts reduces larval survival in response to axenic worms. Interestingly, *Drosophila neotestacea* flies carrying *Spiroplasma* show delayed mortality when parasitized with *Howardula aoronymphium* nematodes; however, *Wolbachia* does not participate in the survival response to these nematodes [55,56]. Similarly, the presence of *Wolbachia* in *Aedes pseudoscutellaris* has no effect on the mosquito survival to *Brugia pahangi* filarial nematodes [57]. Our current results indicate that the effect of *Wolbachia* alone or together with *Spiroplasma* on *S*. *carpocapsae* during infection of *D*. *melanogaster* larvae depends on the presence or absence of the mutualistic *X*. *nematophila* bacteria in the nematode parasites.

We then investigated whether infection of *D*. *melanogaster* with *S*. *carpocapsae* alters the number of endosymbiotic bacteria in the infected larvae. Although compared to larvae not exposed to nematodes, *Spiroplasma* numbers remain unaffected in W+S+ larvae upon infection with *S*. *carpocapsae*, infection with either type of nematode (symbiotic or axenic) reduces *Wolbachia* numbers in W+S+ and W+S- *D*. *melanogaster* larvae. This could imply that *Wolbachia*, but not *Spiroplasma*, forms a target for *S*. *carpocapsae* pathogenesis. In agreement with the current findings, we have found previously that infection of *D*. *melanogaster* adult flies with *Photorhabdus luminescens*, the mutualistic bacterium of the entomopathogenic nematodes *Heterorhabditis bacteriophora*, has no effect on S*piroplasma* numbers [42]. Interestingly, *P*. *luminescens* infection caused a reduction in *Wolbachia* numbers in flies carrying this endosymbiont only. This suggests that certain entomopathogenic nematodes and their mutualistic bacteria employ currently unknown strategies to interfere with the growth of endosymbionts in certain insect hosts. Microbial infection can also increase endosymbiont numbers in *D*. *melanogaster*, as demonstrated by the rise of *Spiroplasma* in flies infected with *Micrococcus luteus* or *Erwinia cartovora* [46]. Together these findings indicate species-specific interactions between exogenous microbes and endosymbiotic bacteria in *D*. *melanogaster*.

Previous studies have also estimated the impact of endosymbionts on pathogen load in infected flies and found that the presence of *Wolbachia* does not influence the replication of *Pseudomonas aeruginosa* [54]. Similarly, we have observed recently that the presence of endosymbionts in *D*. *melanogaster* adult flies do not affect *P*. *luminescens* numbers [42]. Our current data are in agreement with these findings since we also find no changes in *X*. *nematophila* cell numbers in any of the strains used in the experiments, suggesting that the growth of this entomopathogenic bacterium is independent of the presence of *Wolbachia* alone, the simultaneous presence of *Wolbachia* and *Spiroplasma*, or the absence of both endosymbionts in *D*. *melanogaster*.

The transcriptional induction of genes encoding antimicrobial peptides or other effector molecules serves as an indicator of immune signaling activation in *D*. *melanogaster* [58]. We have investigated whether endosymbionts in *D*. *melanogaster* larvae can affect the induction of immune-related genes in the context of nematode infection. Our results demonstrate that the absence of both endosymbionts upregulates the Toll pathway in response to *S*. *carpocapsae* axenic nematodes. It was previously shown that infection with *H*. *bacteriophora* axenic nematodes also upregulated *Drosomycin* transcript levels compared to symbiotic nematodes in *D*. *melanogaster* flies [6]. These results show that induction of Toll signaling is not specific to *S*. *carpocapsae* nematodes only. In addition, failure of *S*. *carpocapsae* symbiotic and axenic nematodes to provoke immune gene upregulation in larvae containing *Wolbachia* only or both *Wolbachia* and *Spiroplasma* suggests a potential interference with the activation of immune signaling in response to nematode attack. The nature of the molecular mechanism through which endosymbionts might interact with the *D*. *melanogaster* immune signaling during nematode infection requires further investigation.

PO is the primary enzyme that regulates melanization at the wound sites and around invading microbes in the hemolymph [59]; however, *X*. *nematophila* bacteria released from *S*. *carpocapsae* nematodes can suppress the melanization response [10]. Here we show that in the absence of both *Wolbachia* and *Spiroplasma*, *S*. *carpocapsae* nematodes and their associated *X*. *nematophila* bacteria fail to suppress the PO activity in *D*. *melanogaster* larvae, but they are able to suppress the activity of the enzyme either in the presence of *Wolbachia* only or for both endosymbionts. This implies that the suppression of PO activity by this nematode-bacteria complex is strongly dependent on the presence of *Wolbachia* and probably *Spiroplasma* endosymbiotic bacteria. Of note, the introduction of certain *Wolbachia* strains into *D*. *melanogaster* and *Drosophila simulans* flies as well as *Aedes aegypti* mosquitoes triggers hemolymph melanization in the absence of infection with exogenous pathogenic microbes [60].

Dietary macronutrients are one of the essential factors that promote host-endosymbiont interactions [61] and that the host metabolism may be altered in the presence of endosymbionts and in the context of nematode infection. *D*. *melanogaster* flies carrying *Wolbachia* have elevated insulin signaling [62], and in *Brugia malayi* that contain *Wolbachia*, the endosymbiont relies on the nutrients (glucose) and energy stores (glycogen) of its host filarial nematode [63]. Here we show that *D*. *melanogaster* larvae carrying *Wolbachia* have increased levels of glycogen and trehalose, whereas levels of triglyceride and glucose are unchanged. Our results are consistent with the notion that these endosymbionts confer little to no beneficial fitness effect to their host. *Spiroplasma* also relies on host lipid availability for its own proliferation [64]. Lipid metabolism and storage in *D*. *melanogaster* occurs in lipid droplets, which are mainly localized in the fat body tissue, although recent evidence indicates that lipid droplets perform additional functions through interactions with pathogenic microbes [65]. We show that the presence of both *Wolbachia* and *Spiroplasma*, but not *Wolbachia* alone, increases the number and size of lipid droplets in the fat body, suggesting increased lipid accumulation in the fat body. We also show that larvae carrying or lacking both endosymbionts have increased lipid droplet size upon symbiotic nematode infection, which correlates with higher levels of triglycerides, whereas infection with axenic nematodes has the opposite effect. These results suggest that despite the presence or absence of *Wolbachia* and *Spiroplasma*, *X*. *nematophila* mutualistic bacteria may affect fatty acid concentrations during infection; however, these changes in the host do not promote pathogen replication.

In spite of recent advances in the insect innate immunity field, our understanding of the role of endosymbiotic bacteria in the host immune response to entomopathogenic nematode infections remains largely unexplored. Results from the research presented here will improve our understanding of the complex symbiotic interactions between eukaryotic hosts and microbial organisms in the context of parasitic infections. From the practical point of view, a better understanding of insect-endosymbiont relationships could potentially lead to the development of alternative strategies for the efficient management of agricultural insect pests and vectors of human diseases.

## Materials and methods

### Fly stocks

*Drosophila melanogaster* third instar larvae carrying both *Wolbachia pipientis* (strain *w*Mel) and *Spiroplasma poulsonii* (strain MSRO, designated as W+S+), no endosymbiotic bacteria (W-S-), or *Wolbachia* only (W+S-) were used in all experiments, as previously described [42]. All fly strains were amplified for experimentation with approximately 2.5 g of Carolina Formula 4–24 Instant *Drosophila* media (Carolina Biological Supply, USA), 10 ml of deionized water, and a few granules (approximately 0.003 g) of dry baker’s yeast. All fly strains were grown at 25°C and a 12:12-hour light:dark cycle.

### Nematode stocks

The entomopathogenic nematode *Steinernema carpocapsae* harboring the Gram-negative bacteria *Xenorhabdus nematophila* (symbiotic nematodes) were amplified in the larvae of the wax moth *Galleria mellonella*. Nematodes lacking *X*. *nematophila* (axenic nematodes) were generated as described [8]. Prior to use, axenic nematodes were surface sterilized in 1% bleach and washed five times with sterile distilled water to remove any residual bacteria from their surface. Infective Juvenile (IJ) stage nematodes 2–4 weeks old were used in all experiments.

### Larval survival

To each well of a 96-well plate (Corning), 100 μl of 1.25% agarose were added. Sterile water (10 μl) suspensions containing 100 *S*. *carpocapsae* symbiotic or axenic nematodes were transferred to each well together with an individual *D*. *melanogaster* third instar larva. To remove any food particles from the cuticle, each larva was washed with sterile distilled water prior to infection. The wells were covered with a Masterclear real-time PCR film (Eppendorf, USA) and two holes were pierced for ventilation. For control treatment, 10 μl of sterile water were applied to each larva and survival was monitored every 12 h for up to 108 h post-infection. Twenty larvae per strain per treatment were used and the experiment was repeated three times.

### Endosymbiont numbers

Four larvae from each fly strain were infected with *S*. *carpocapsae* symbiotic or axenic nematodes and subsequently frozen at 12, 36 and 60 h post infection. DNA samples were extracted from the frozen larvae using DNeasy Blood and Tissue kit (Qiagen) following the manufacturer’s protocol. For estimation of endosymbiont load, all DNA samples were normalized to 300 ng. Quantitative PCR was performed in twin-tech. semi skirted- 96 well plates (Eppendorf) in a Mastercycler^®^ ep realplex^2^ (Eppendorf). The experiments were repeated three times and samples were run as technical duplicates. *Wolbachia* and *Spiroplasma* CFUs were determined using the standard curves generated using plasmid DNA and PCR conditions were followed as described [42]. Relative numbers of *Wolbachia* and *Spiroplasma* cells were determined as a ratio of the endosymbiont number in larvae infected with *S*. *carpocapsae* symbiotic or axenic nematodes and in control larvae treated with water.

### *Xenorhabdus nematophila* standard curve

DNA from *X*. *nematophila* bacteria was extracted using the Invitrogen™ Ambion™ TRIzol™ Reagent. PCR amplifications were performed using the *X*. *nematophila* 16S rRNA primer sequences (Table 1). The cycling protocol used was described [42]. The samples were run as technical duplicates. Standard curve for *X*. *nematophila* 16S rRNA was used to estimate bacterial load in infected larvae using the same method as described before [42].

**Table 1:** Primer sequences and annealing temperatures used for quantitative RT-PCR (qRT-PCR).

Gene	Accession No	Primer	Sequence (5'-3')	Tm (°C)
*Diptericin*	CG10794	Forward Reverse	TGCGCAATCGCTTCTAC GTGGAGTGGGCTTCATG	56
*Drosomycin*	CG10810	Forward Reverse	TGAGAACCTTTTCCAATATGATG CCAGGACCACCAGCAT	56
*Turandot-A*	CG31509	Forward Reverse	AGATCGTGAGGCTGACAAC CCTGGGCGTTTTTGATAA	61
*Puckered*	CG7850	Forward Reverse	GGCCTACAAGCTGGTGAAAG AGTTCAGATTGGGCGAGATG	61
*RpL32*	CG7939	Forward Reverse	GATGACCATCCGCCCAGCA CGGACCGACAGCTGCTTGGC	61
*X*. *nematophila 16s rRNA*		Forward Reverse	GCTTGCTGTTTTGCTGACGA CCGAAGGTCCCCCACTTTAC	61

### *Xenorhabdus nematophila* quantification

Four larvae from each fly strain were infected with *S*. *carpocapsae* symbiotic IJs and frozen at 12, 36 and 60 h post infection. DNA samples were extracted from the frozen larvae using the Invitrogen™ Ambion™ TRIzol™ Reagent. DNA samples (300 ng) were used in a total reaction volume of 20 μl. The cycling protocol was the same as described [42]. *X*. *nematophila* CFUs were calculated using the standard curve. The experiment was repeated three times.

### Immune gene signaling

Four larvae from each fly strain were infected with *S*. *carpocapsae* symbiotic or axenic IJs and frozen at 12, 36, and 60 h after infection. Controls were treated with water. Total RNA was extracted using the PrepEase RNA spin kit (Affymetrix USB) following the manufacturer’s instructions and adjusted to 300 ng. Complementary DNA (cDNA) synthesis and qRT-PCR were performed as described [42]. Primers were purchased from Eurofin MWG Operon (Table 1). Relative gene transcript levels are calculated relative to the housekeeping ribosomal gene, *RpL32*, and expressed as a ratio compared to mRNA values of uninfected control samples. The experiment was repeated three times and values represent mean and error bars show standard deviations.

### Melanization and PO response

For assessing melanization, 10 larvae from each strain were heat treated to visualize blackening of the crystal cells, as described previously [52]. For estimating PO activity, larvae from each fly strain were infected with 10 *S*. *carpocapsae* symbiotic or axenic nematodes. Larvae were collected 24 h post infection, washed with 1X cold PBS and hemolymph was collected in 2.5X protease inhibitor (Sigma) by puncturing the larvae with a needle. The hemolymph was then loaded onto a spin column (Pierce, ThermoFisher) and spun at 13,000 rpm at 4°C for 10 min. Protein concentrations were estimated using a BCA test (Pierce, ThermoFisher) and a mixture of 15 μg of protein (diluted in 2.5X protease inhibitor) with 5 mM CaCl_2_ was added to L-DOPA (15 mM in phosphate buffer, pH 6.6) for a final volume of 200 μl. The samples were measured at absorbance 492 nm after 34 min of incubation at 29°C in the dark and compared to a blank. Each experiment was run in technical duplicates and repeated three times.

### Metabolic activity

Ten-fifteen larvae from each fly strain were infected with 10 *S*. *carpocapsae* symbiotic or axenic nematodes, or treated with sterile distilled water and larvae were collected 24 h later. Samples were processed using a previously published protocol [66]. Protein quantification was performed using the Pierce^TM^ BCA protein assay kit (ThermoFisher Scientific) following manufacturer’s instructions. The plate containing the samples and BCA reagents was covered and placed in a 37°C incubator for 20 min. Absorbance was measured at 562 nm and protein concentrations of samples were calculated from the standard curve.

For estimating triglycerides in nematode-infected and uninfected larvae, samples were diluted 1:1 in PBS-Tween to which 200 μl of the Infinity^TM^ Triglycerides Liquid Stable Reagent (ThermoFisher Scientific) had been added. The 96-well plate containing the samples was covered and incubated at 37°C for 30 min, and absorbance was measured at 540 nm. Standard curve was generated using the Glycerol Standard Solution (Sigma) and Triglyceride content in samples was calculated using the glycerol standard curve.

For estimating trehalose levels in the larvae, samples were diluted 1:8 in Trehalase Buffer (5 mM Tris pH 6.6, 137 mM NaCl, 2.7 mM KCl). Free glucose was estimated from samples diluted in Trehalase Buffer (TB) whereas trehalose content was calculated from samples digested in Trehalase Stock (3 μl of porcine trehalase in 1 ml of TB) by subtracting the amount of free glucose from the standard curve.

For estimating glucose and glycogen levels, samples were diluted 1:3 in PBS. Samples were further divided into two sets; the first set was diluted 1:1 in amyloglucosidase stock (1.5 μl of amyloglucosidase in 1 ml of PBS) and the second set was diluted 1:1 in PBS. Samples (30 μl) from each set were added to individual wells of a 96-well plate and allowed to incubate at 37°C for 60 min. To each well, 100 μl of HK (Glucose Assay Reagent, Sigma) were added and absorbance was measured at 340 nm after 15 min at room temperature. The amount of glucose was calculated from the samples in PBS using the glucose standard curve. For glycogen, the absorbance of glucose in PBS was subtracted from the absorbance of the samples digested with amyloglucosidase (Sigma). Glycogen content was calculated from the glycogen standard curve.

For all metabolic assays, each experiment was run in technical duplicates and repeated four times. The amounts of triglyceride, trehalose, glucose and glycogen are expressed relative to the total protein content in each sample.

### Lipid droplet (LD) staining

Ten larvae from each fly strain were infected with 10 *S*. *carpocapsae* symbiotic or axenic nematodes and samples were collected 24 h post-infection. Dissections were performed in 1X PBS, and fat body tissues were separated from the rest of the larval carcass. They were then fixed in 4% paraformalydehyde prepared in PBS for 30 min at room temperature followed by rinsing with 1X PBS twice. They were then incubated in the dark for 30 min in 1:1000 dilution of 0.05% Nile Red in 1 mg/ml of methanol. These tissues were then mounted in ProLong^TM^ Diamond AntiFade Mountant with DAPI (Life Technologies). Images were obtained using a Confocal Olympus FluoView^TM^ FV1000 imaging system. Data were collected from fat body tissues of each of the 10 larvae. LD area was assessed using ImageJ software (National Institutes of Health). A minimum of five random regions were selected for LD size quantification from each fat body tissue.

### Statistical analysis

All values were expressed as means ± standard deviation. Survival experiments were analyzed using a log-rank (Mantel-Cox) and Chi square tests. Bacterial load and endosymbiont numbers were analyzed using unpaired two-tailed *t-*test. Gene expression, PO activity, metabolic activity and lipid droplet sizes were analyzed using one-way analysis of variance (ANOVA) with a Tukey post-hoc test for multiple comparisons. All figures were generated using GraphPad Prism7 software.
